# Evaluating a community-based early childhood education and development program in Indonesia: study protocol for a pragmatic cluster randomized controlled trial with supplementary matched control group

**DOI:** 10.1186/1745-6215-14-259

**Published:** 2013-08-16

**Authors:** Menno Pradhan, Sally A Brinkman, Amanda Beatty, Amelia Maika, Elan Satriawan, Joppe de Ree, Amer Hasan

**Affiliations:** 1 Amsterdam Institute for International Development, Faculty of Economics and Business Administration, VU University Amsterdam, De Boelelaan 1105, Amsterdam 1081 HV, The Netherlands; 2 Faculty of Economics and Business Administration, University of Amsterdam, Amsterdam 1012 WX, The Netherlands; 3 Telethon Institute for Child Health Research, Centre for Child Health Research, University of Western Australia, Perth 6907, Australia; 4 School of Population Health, Discipline of Public Health, The University of Adelaide, Adelaide 5005, South Australia; 5 Mathematica Policy Research, 600 Alexander Park, Princeton NJ 08540, USA; 6 Department of Sociology, Gadjah Mada University, Jalan Bulaksumur, Yogyakarta 55281, Republic of Indonesia; 7 Department of Economics, Gadjah Mada University, Jalan Bulaksumur, Yogyakarta 55281, Republic of Indonesia; 8 National Team for the Acceleration of Poverty Reduction, Office of Vice President, Jl. Kebon Sirih Raya No.35, Jakarta Pusat 10110, Republic of Indonesia; 9 Education Unit, Human Development Department, World Bank, Jl. Jenderal Sudirman, Jakarta 12190, Republic of Indonesia

**Keywords:** Early childhood education and development, Indonesia, Impact evaluation

## Abstract

**Background:**

This paper presents the study protocol for a pragmatic cluster randomized controlled trial (RCT) with a supplementary matched control group. The aim of the trial is to evaluate a community-based early education and development program launched by the Government of Indonesia. The program was developed in collaboration with the World Bank with a total budget of US$127.7 million, and targets an estimated 738,000 children aged 0 to 6 years living in approximately 6,000 poor communities. The aim of the program is to increase access to early childhood services with the secondary aim of improving school readiness.

**Methods/Design:**

The study is being conducted across nine districts. The baseline survey contained 310 villages, of which 100 were originally allocated to the intervention arm, 20 originally allocated to a 9-month delay staggered start, 100 originally allocated to an 18-month delay staggered start and 90 allocated to a matched control group (no intervention). The study consists of two cohorts, one comprising children aged 12 to 23 months and the other comprising children aged 48 to 59 months at baseline. The data collection instruments include child observations and task/game-based assessments as well as a questionnaire suite, village head questionnaire, service level questionnaires, household questionnaire, and child caretaker questionnaire. The baseline survey was conducted from March to April 2009, midline was conducted from April to August 2010 and endline conducted early 2013. The resultant participation rates at both the district and village levels were 90%. At the child level, the participation rate was 99.92%. The retention rate at the child level at midline was 99.67%.

**Discussion:**

This protocol paper provides a detailed record of the trial design including a discussion regarding difficulties faced with compliance to the randomization, compliance to the dispersion schedule of community block grants, and procurement delays for baseline and midline data collections. Considering the execution of the program and the resultant threats to the study, we discuss our analytical plan and intentions for endline data collection.

**Trials registration:**

Current Controlled Trials ISRCTN76061874

## Background

Early childhood is a very active period of brain development that lays the foundation for later learning. Research shows that a child’s early life has consequences for their adult years [1]. It is similarly recognized that many of the problems arising in early childhood have associated social and financial costs that cumulatively represent a considerable drain on a country’s resources [2]. A country’s future productivity may be undermined if children are not protected and afforded the opportunities to thrive. In addition, the deleterious effects of poor outcomes in early childhood can be long-lasting, affecting school attainment, employment, wages, criminality and social integration. It is more cost-effective to institute preventive measures and support for children early on than to compensate for disadvantage as they grow older [3].

Strong foundations including good health, nutrition and a nurturing environment during childhood can help ensure a smooth transition to primary school, a better chance of completing basic education, and a route out of poverty and disadvantage [4]. School readiness leads to school success; however, the characteristics of school readiness are multi-dimensional. Early predictors of school success point to the contribution of positive peer relationships, and sensitive and stimulating family processes [5]. The literature suggests a link between children’s learning-related social skills and academic performance. For example, a child’s social adjustment to kindergarten has been linked to the child’s performance and involvement in the school [6,7], and children’s work-related skills (for example, the degree to which children show compliance with instructions) are closely related to school success as well as their self-regulation [8]. Although many of these skills increase with age, there is enough variation in how children develop during their first 5 years of life to suggest that chronological age is not an effective indicator of school readiness in itself [9]. School outcomes, especially achievement, remain remarkably stable after the first years of school [10,11], once again indicating the importance of health and holistic child development prior to school entry.

### Early childhood development and education in developing countries

The Strong Foundations Education for All Global Monitoring Report [4] conducted by UNESCO states that there are large disparities in educational attainment within countries. Developing countries with a rapidly increasing divide between rich and poor are particularly vulnerable to creating a generation of poor and uneducated children that will eventually undermine the countries’ increasing prosperity [12]. Children from poorer and rural households and those socially excluded have significantly less access to early childhood care and education than those from richer and urban households [13].

Although there is a wealth of data in the medical, sociological and economic academic literature on the health and nutritional status of young children in developing countries, there is comparatively little data on early childhood development and school readiness (for example, physical development, language and cognitive development, communications skills, and socio-emotional development). It is generally believed that low parental education levels play a causal role in explaining poor child health status in developing countries, but it is not clear if this also applies to other dimensions of skill formation in early childhood [3]. Inadequate knowledge by parents about the importance of early childhood and the long-term impact of early parenting practices may all contribute to low levels of parental investment in their children’s early skill formation [3].

*The Lancet* has published two series [13,14] of journal articles on child development in developing countries. The articles pull together the available literature on early child development in developing countries and note that the primary causes of poor development include: malnutrition, iodine and iron deficiency, inadequate stimulation, malaria, violence, maternal depression, exposure to heavy metals, diarrhea, and HIV/AIDS [15]. The second paper [14] assesses strategies to promote child development and prevent the loss of developmental potential. The authors’ findings suggest that the most effective interventions are those that provide direct learning experiences to the children and families, are targeted towards the youngest and most disadvantaged, are of adequate duration, are high quality and high intensity, and are integrated with family support, health, nutrition and/or educational systems and services. Of the research available, findings indicate that an approach that combines nutrition, health, care and education is more effective in improving young children’s current welfare and their development rather than limiting interventions to one aspect [16-18]. A challenge, however, is that traditional midwives, health workers, early child care and early education staff in developing countries typically have minimal education themselves, and are often relatively poorly remunerated [4,19].

With a greater scale for improvement in school readiness outcomes, the evaluation of Early Childhood Education and Development (ECED) programs in developing countries affords a greater scope for investigation into the facilitators and barriers for success. Considering the relative lack of published literature on the impact of early childhood and education programs in developing countries, it is imperative that such programs are evaluated in a way that can contribute to the international literature to advise and inform governments and funding bodies.

### Indonesian context

Indonesia continues to show economic growth despite the recent global financial crisis and is currently classified as a lower to middle income country [20]. Such economic growth should provide the people of Indonesia improved living conditions. However, the poverty rate still sits at 12.2% and with a population greater than 225 million this equates to over 27 million people living below the poverty line [20,21]. In addition, it is estimated that up to half the population are vulnerable to poverty with the inequality between rich and poor vast [22], and given the size and varying conditions across Indonesia, regional disparities are a fundamental feature of poverty in the country [23,24].

In Indonesia, the use of maternal child health services has increased over the last 15 years and the proportion of births attended by a professional health provider has also increased dramatically to nearly 75%; and there has been a corresponding reduction in maternal and infant mortality rates. The maternal mortality rate dropped from 423 in 1980 to 229 per 100,000 in 2008 [25,26] and the infant mortality rate dropped from 56 in 1990 to 31 per 1,000 in 2008 [21]. However, there is still weakness in Indonesia’s provision of health services and financial constraints on people’s ability to access those services that do exist [25] and thus, despite the gains, maternal and infant mortality rates remain the highest in East Asia [20,25].

A large disparity in socio-economics is also reflected in the wide variations in educational outcomes [27]. ECED programs include services for children from birth through to the age of 6 years. Provided under different auspices and settings, these services promote many different aspects of young children’s development and learning, with some services more educationally-focused and others emphasizing physical care, health or nutrition. Typically, ECED programs may include group programs (preschools, kindergartens and child care centers), home-based day care programs (sometimes known as family child care), and home visiting or parent education and support programs.

In Indonesia, in 2004, there were four different kinds of educational programs available for children aged 0 to 6 years: kindergarten (47,696 programs), Islamic kindergarten (1,560), playgroups (5,169) and childcare centers (1,789) [28]. Of the 28 million children aged 0 to 6 years in Indonesia, only a small minority participated in early childhood education services prior to primary school, which children are expected to start at the age of 7 years. Enrolment was estimated to be only 8% whereas the global average enrolment rates for low income countries stood at 24% [28]. In 2004, 85% of children in the poorest quintile (measured using household per capita consumption) were not participating in any kind of early childhood programs. In addition, children from the poorer villages start school later, complete fewer years of schooling, and have higher dropout and repetition rates [22].

### The early childhood education and development (ECED) program

The overarching objective of the program is to improve poor children’s overall development and readiness for further education within a sustainable quality ECED system. To achieve this objective, the project aims to:

1) Increase the capacity of poor communities to engage in participatory planning that will result in new or improved ECED services for their children and families.

2) Prepare the foundation for a sustainable ECED system through budgetary commitments from participating districts, establishment of a national quality assurance and professional development system, and district capacity building.

3) Ensure continuous improvement of service delivery and system building through establishing effective project management, and monitoring and evaluation.

The project targets an estimated 738,000 children aged 0 to 6 years and their parents/caretakers living in approximately 6,000 poor communities located in 3,000 villages within 50 poor districts throughout Indonesia. Particular attention was to be given to children aged 2 to 4 years as some children may enroll in kindergarten by the age of 5 years. The project is additionally expected to have a demonstration effect on the Government, which may then expand the coverage of ECED services for poor children in Indonesia [28].

This project, implemented from 2006 to 2012, is financed through a credit from International Development Assistance by the World Bank and a grant from the Government of the Kingdom of the Netherlands. Such a loan is particularly applicable to the ECED sector in Indonesia given that it is at the ‘start-up stage of developing a system and that it requires major technical inputs’ [28]. The project costs a total of US$127.7 million (International Development Association funding from the World Bank equaling US$67.5 million, the Netherlands grant equaling US$25.3 million and the Government of Indonesia US$34.9 million).

The original loan agreement and project appraisal document specified the requirements for achieving each of the project aims. Of primary importance to this study and the basis on which the trial was first designed was the detail in these documents regarding the intended delivery of the program. Fifty districts were selected to participate in the project according to certain criteria including: low participation rates of children in ECED services, poverty, commitment to developing an ECED agenda, existence of staff with a mandate for managing ECED, existence of governance structures to support the integration of early education and health services, and readiness to finance some of the project activities to maximize the potential for sustainability after the program funding ceases.

After the 50 project districts were selected, 60 villages each with the greatest need for ECED services (selected by the highest number of children aged 0 to 6 years, proven interest in receiving the project and high poverty rates) were identified and targeted to receive the project. While oversight and coordination happened at the district and village levels, services were to be delivered most intensively in two dusuns (a dusun is a small community or sub-village within the broader village). However, it was expected and hoped that all children throughout a village would utilize services concentrated in the two target dusuns. Typically, Indonesian villages consist of about five dusuns, each with approximately 60 children aged 0 to 6 years, which was considered an optimal size for service delivery (that is, those with the highest numbers of poor households and children aged 0 to 6 years). Services were not to be delivered to all 60 villages simultaneously but rather rolled out in three waves (called ‘batches’) of 20 villages each, 9 months apart.

In order to deliver services, each village received a grant of approximately US$9,000, disbursed in tranches over 3 years and shared among the two target dusuns. Grant funds were delivered to a village level account supervised by a village management team, also responsible for project oversight and coordination. Implementation was also driven by one teacher and one community development worker (CDW) per dusun, the latter focusing on outreach to children aged 0 to 3 years and their families. These two staff members received district level training in early child development, nutrition and community-driven development provided by the project.

With support from project leadership, each dusun was to allocate grant funds to a menu of ECED service options. Services for older children (aged 3 to 6 years) were expected to be center-based, while services for families with young children (birth to 3 years) could include a combination of group-based services (for example, parent education, especially in regard to health and nutrition; early learning and stimulation) and individual services (for example, home visiting). Requirements related to the use of the funds were that villages must: i) use the funds to enhance or expand existing services; ii) plan ways to increase the number of poor children and families served, and to improve the quality of community programs; and iii) provide services in compliance with a set of essential standards including health and safety provisions. Within those requirements, villages had choices in the specific scheduling, distinctive features, implementation approaches and physical settings in which services were delivered. No land acquisition or resettlement was to be undertaken as communities were expected to utilize and enhance existing spaces/facilities, or coordinate with other local level projects to fund new facility construction. Further information about the program is detailed in the Project Appraisal Document [28].

In addition to the program delivery, the sector investment loan included funds for monitoring, supervision and evaluation of the program to be undertaken jointly with the Ministry of National Education (MoNE) and the World Bank. One primary facet of this agreement was an impact evaluation with the goal of understanding whether the project improves children’s development and readiness for primary school, and what factors (for example, community cohesion, remoteness and parents’ socio-economic status) contribute to the effectiveness of ECED services.

This paper documents the study protocol for this evaluation consistent with the guidelines for the reporting of a randomized controlled trial (RCT) and, in particular, the extension of the Consolidated Standards of Reporting Trials (CONSORT) statement for cluster randomized trials [29] and pragmatic RCTs as advised by Zwarenstein *et al*. [30].

### Aims

#### Objectives of the research

The agreement between the Early Childhood Development Unit (ECDU) in the National Ministry of Education and the World Bank outlines joint research with the objectives:

1) To ensure a high quality impact evaluation of the ECED project that uses appropriate and state of the art methods to measure child development, and has a rigorous design to ensure that causal effects can be determined.

2) To conduct analysis based on the data generated by the impact evaluation surveys to: i) establish the impact of the ECED program on early childhood development outcomes; and ii) obtain greater insight into the patterns and correlates of child development in Indonesia.

3) To raise awareness and stimulate discussion among policy makers and the general public regarding the importance of early childhood development and effective approaches to improving them.

This agreement further notes that the objectives are of strategic relevance, both for the Indonesian Government and the World Bank, for the following reasons:

1) The evaluation will provide credible information on whether the community-based approach now being implemented by the Government positively impacts child development outcomes and, if so, the pathways by which it does.

2) As the first-ever survey to measure early child development using a broad range of dimensions in Indonesia, the analyses will give greater insight into the factors that influence early child development in Indonesia.

3) By undertaking such a comprehensive and rigorous study, it is hoped that the results will provide the evidence required to raise awareness and advocate for the early years and the importance of school readiness.

##### Study hypotheses

The overarching goal of the ECED program is to improve poor children’s overall development and readiness for further education within a sustainable quality ECED system. However, for the purposes of the trial we required more specific aims and thus developed the following hypotheses. Relative to the non-intervention group, participants in the experimental group of the study will:

1) Have greater access to ECED services.

2) Have a higher participation rate in ECED services.

3) Have a higher enrolment rate in school at earlier ages.

4) Be more ‘school ready’.

5) Have higher community awareness about the importance of ECED.

6) Have higher persistent breastfeeding rates, improved nutrition and improved early childhood stimulation.

## Methods/Design

### Trial design

The trial is a pragmatic cluster (by village) RCT with an additional matched control group. A clustered design was necessary as the program was implemented at a village level and thus individual recruitment was not sensible due to the potential for contamination. This design also allowed for a pragmatic evaluation of how the program is implemented by communities. As the aim is for the ECED initiatives to be sustainable after the period of the loan, it is additionally important to understand how communities implement the program from a process point of view.

As mentioned above, to facilitate the implementation of the ECED project, the 60 project villages per district were allocated to three implementation batches. It was planned that Batch 1 would receive the first block grants at the start of the project, and block grants for Batch 2 and Batch 3 were to follow after 9 and 18 months, respectively. The evaluation design made use of this feature of the implementation: a selection of villages was randomly allocated to either Batch 1 or Batch 3 (within each district).

### Recruitment

The project as a whole covers 50 districts across Indonesia. By the time the MoNE expressed support for a randomized evaluation design, many districts had already decided when each of the 60 project villages would receive funding (that is, districts had already allocated villages to a particular batch), making it too late to implement a randomized roll-out of services in these districts. Districts that had not yet decided when beneficiary villages would receive the project were contacted by the MoNE and asked about their willingness to randomly allocate 20 villages out of 60 to Batches 1 and 3. As batch implementation was to begin 9 months apart, it was anticipated that there would be 18 months between Batches 1 and 3. In deciding upon the final ten districts to participate in the study, the MoNE made an effort to ensure geographic diversity with the goal of producing a sample that broadly represented project districts. These districts were: Sarolangun, Rembang, Gorontalo, Kulon Progo, Sidenreng Rappang (Sidrap), Majalengka, Ketapang, North Bengkulu, Middle Lombok and East Lampung. Random selection at the district level would of course have been preferable and allowed for districts to have been representative of the project, but this was impossible due to timing and lack of willingness of districts to randomize.

Randomization of the 20 villages took place through a public lottery in each district, supervised by MoNE staff. The first ten villages selected were allocated to Batch 1, treatment, and the latter ten were allocated to Batch 3, control (which were to receive treatment 18 months later).

#### Matched control sample

Due to concerns that the randomization and/or planned timelines of implementation would not be adhered to, the research design also incorporated ten matched control villages per district. The matched controls were identified by asking district officials to identify ten control villages that were not part of the ECED project (that would never receive the treatment), but otherwise were similar to the ten randomly selected treatment Batch 1 villages. The rationale for two control groups was practical – to reduce the risk of not being able to identify impact because of unforeseen implementation challenges. It was also technical – 18 months (the anticipated gap between Batch 3 and Batch 1) was thought to be potentially insufficient to detect significantly different child development outcomes, and thus the matched controls were to allow for comparison over 3 years between baseline and endline rather than just the 18 months with the randomized control. One potential implementation challenge, as is common during implementation, was that the project would start late and accelerate as implementation progressed. This would mean that less than 18 months may elapse before controls received the treatment. Thus, we included the matched controls, which aimed to make the study less vulnerable to changes in timing and enabled tracking of longer-term impacts. In sum, the project design was to make use of one treatment group of 100 villages and two control groups, each consisting of 100 villages, thus 300 sample villages in total.

#### Deviations from original random assignment

**Figure 1:**
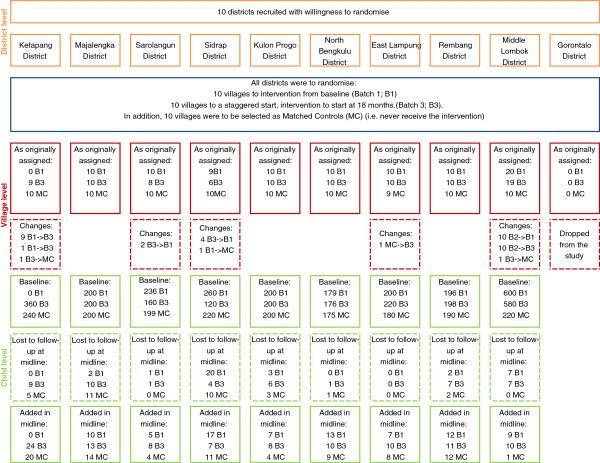
Participant flow diagram.

In December 2008, before the baseline survey had commenced, but simultaneous with the treatment villages receiving their grant (but not before services began), a World Bank team uncovered that five districts had not complied with the village level randomization. Specifically, this meant that these districts took action to implement the project in an order that was different to what was originally agreed to with the MoNE and the World Bank in 2006. The main reason stated by the districts for their lack of compliance to the randomization was convenience – they felt it was easier to roll-out the project to villages that were proximate rather than complying with the randomization design which did not allow for clustering project sites. The districts that did not comply are shown in the participant flow diagram (Figure 1) with the number of villages that remained in the original batch to which they were assigned. Note that full compliance would mean ten villages in each group and no changes.

This alteration of the implementation plan is of course a serious concern for the evaluation as it compromises the design and in turn the quality of the information that the study will be able to generate. The impact evaluation team decided to drop the survey work in the district (Gorontalo) where noncompliance was the most serious. The rationale for this change was that external validity was already compromised due to the fact that the ten districts chosen for the evaluation were not randomly chosen from the 50 project districts, but were chosen because they agreed to participate in the evaluation. The team also felt that with such poor compliance in Gorontalo there would be little possibility of exploiting the randomized design in the analyses.

In the district of Middle Lombok , all 60 project villages (not just 20 as in the other nine districts) were randomized to the staggered rollout. This was the independent choice of the district, not the study team. Therefore, the sample of 30 villages that were originally allocated to Gorontalo were replaced by villages in East Lombok and all 60 project villages in East Lombok were included in the study sample, thus increasing the total sample size by ten villages, to 310. The rationale for including all 60 villages was that it would offer more power. Additionally the inclusion of Batch 2 villages may provide some information about different periods of exposure to treatment.

Additionally, due to compliance issues, some of the villages selected as matched controls in 2006 were changed at the time of baseline in 2008. Due to the matching strategy, which involved pairing the treatment villages with individual similar villages, if treatment (Batch 1) villages changed, we also needed to newly identify the most appropriate matched control.

### Sample size calculation

The average treatment effects at the village level, using a cluster randomized design, drove the sample size calculations for this experiment. It was challenging to estimate baseline variations between and across clusters for the outcome measures and to set minimum detectable effect sizes as a project of this kind was unprecedented in Indonesia, and none of the intended outcome measures had ever been collected in the country. Thus, the research team used a combination of education measures in Indonesia and ECED measures from a similar project in a country with comparable socioeconomic indicators, the Philippines.

In order to estimate changes in comparable outcome measures, the team calculated the intracluster correlation using net enrolment ratios in junior secondary education as observed in the 2004 Indonesian national household survey (Susenas). We used secondary rather than primary enrolment due to Indonesia’s near universal enrolment at the primary level. In general, one enumeration area in Susenas is a village, and in each area, 16 households are interviewed. The intracluster variation between enumeration areas for junior secondary enrollment was estimated at 0.23, which was used in the subsequent power calculations.

To predict our effect sizes for the sample calculations, we used results from a study conducted in the Philippines on the impact of a comparable, community-based ECED project [31]. The study evaluated an ECED initiative of the Philippine Government using longitudinal data collected over 3 years on a cohort of 6,693 children aged 0 to 4 years at baseline. The Philippine ECED program was especially relevant for this study as the Indonesian program was modeled on the Philippine example. The Philippine ECED program included a range of health, nutrition, early education and social service programs, and aimed to improve the survival and developmental potential of those children who were most disadvantaged and vulnerable. The Philippine ECED project did not introduce new services, but provided a child development worker to each of the program areas to link center-based and home-based services in an integrated multi-sector approach [32].

The Philippines study used outcome measures relating to gross motor skills, fine motor skills, expressive language, receptive language, cognitive skills, self-help and social-emotional development, and reported mostly positive impacts in the range of 0.3 to 1.1 standard deviations [32]. Using these impacts as the minimum detectable effect sizes for the Indonesian study, the minimum number of villages to sample was in the range of 7 to 116 villages, depending on the chosen outcome measure, with half of this sample to be allocated to the treatment group and half to the control. We calculated the minimum number of clusters on the basis of a power of 0.9, with an estimated intracluster correlation coefficient (ICC) of 0.23 and with the requirement of ten children surveyed per village. The Philippines study, however, reported unusually high effect sizes especially when compared to a review (published after the sampling for this study) by Nores and Barnett [33], which suggested an average effect size of 0.255 standard deviations for studies investigating the impact of cash transfers, nutritional, educational or mixed interventions conducted in low and middle income countries. Using this as the minimum detectable effect, keeping all else constant, the minimum number of villages required increases to 182. The study eventually sampled 200 villages randomized into treatment and control groups. The power calculations suggest that this would be more than sufficient to detect impact estimates in similar size to those that are reported for the Philippine ECED intervention, and just sufficient to detect the average effects reported in Nores and Barnett’s review.

The size of the matched control group was set equal to the treatment group. In the analysis, we will also be able to present estimates of the impact that rely on standard difference in difference assumptions, comparing the treatment group with the matched control group. The sample size calculations as discussed above are valid under these assumptions; hence the same sample size for the matched control group.

#### Sampling procedures

The steps taken to obtain the final list of sampled villages are described in the Recruitment section above. The baseline survey contained 310 villages, of which 100 were originally allocated to Batch 1, 20 originally allocated to Batch 2, 100 originally allocated to Batch 3 and 90 allocated to the matched control group. The remainder of this section describes the sampling procedures followed within each village.

In each village, two dusuns were sampled. At baseline it was not clear which dusuns the project would work in and as such the evaluation selected one dusun randomly as well as including the dusun in which the village head’s office was located. The rationale for the selection of the dusun with the village head’s office was that it would have a higher concentration of people and also be a likely location for early childhood services. We felt confident about detecting project participation without yet knowing about project placement as the intervention aimed to have an influence at the village level. On average there are five dusuns per village [28].

Households were randomly selected during the baseline survey from a listing that was created for this evaluation. As individual level enumeration data is not available for Indonesia, the World Bank had to construct a listing. To construct this listing, fieldworkers went to each village, met the village and dusun heads, and asked for a resident roster list of all households by dusun where there was a child aged between 0 and 6 years. In some areas, if the information from the dusun head was incomplete or questionable, this information was cross-checked with records from the posyandu head (integrated child health services clinic) and/or a midwife. In each dusun, survey teams interviewed ten households with children aged 1 and/or 4 years, amounting to 20 households per village and thus 6,200 households in total.

In the case where a sampled dusun had an insufficient number of children in the target ages, field teams randomly selected another dusun, and continued this random selection process until meeting the required 20 households per village. If all the households with children in the target age range as per the listing had been contacted across the entire village, but the survey team still had not interviewed 20 households, then supervisors could ask people in the village if there were other children in the village that met the age criteria (that is, snowballing), and interview them.

The objective of sampling households with children aged 1 and/or 4 years in each village was to follow children who would span the ages of 1 to 7 years during the life of the survey, capturing most of the target age range of the project. Children who were aged 1 year at baseline will have reached the age of 4 years at endline, thus providing an additional comparison with the cohort of children aged 4 years old at baseline. The cohort analysis of the children aged 1 year will provide information on the effectiveness of ECED interventions focused at very early childhood, mostly related to health (nutrition, vaccinations, fine motor skills and gross motor skills) and parenting. Children who were aged 4 years old at baseline will be around 7 years old at endline, and will most likely be enrolled in the early years of primary school. At this point, this cohort will have reached an age where we can assess school performance, and shed light on the project’s impact on increasing school readiness. The analysis based on the older age cohort will provide information on the effectiveness of the ECED interventions aimed at children in the age range of 4 to 6 years old.

#### Midline replacement procedures

Since this study aimed to track the same children over three rounds of data collection, the study team made every attempt to interview the baseline children again at midline in 2010. Across all nine districts, 112 children were not reached in the midline. The most common reasons for a child not being available for midline interview were: the household was impossible to find (17 children), the child had died (nine children), the family refused (five children) or other reasons (81 children).

In the case of children moving, field teams adhered to the following protocol. First, the teams tried to assess whether the child had moved to another village (in the same district) that was in the study sample. If the child had, the child and household were interviewed in the new village. One hundred and one of the children had moved to another village prior to midline, but were able to be identified and interviewed in their new village. Second, if tracking a child to a sample village was impossible, the household was replaced using the same identification procedure from the baseline. The field teams used the dusun level listing from the two sample dusuns, and if this listing did not yield 20 households per village, then the field teams used the procedure outlined above for randomly selecting a new dusun. While replacements were made, the reason for them was not always recorded, and the exact number of children who had moved to another village and were no longer able to be tracked is unknown.

Other reasons for replacing or adding new households included: i) baseline teams had erred in interviewing children in the wrong age group in baseline; or ii) baseline teams were not able to identify 20 households with children who met the age criteria, and thus teams in the midline attempted to search again and add households. While tracking children outside of a sample area might have been possible, the research team decided against it for reasons of cost and questionable benefit to the evaluation. All children who were lost to follow-up at midline were replaced. The participant flow diagram (Figure 1) provides details regarding the number of children lost to follow-up in each study district.

### Outcome measures

As mentioned above, the primary objectives of this study are to establish the impact of the ECED project on early childhood development outcomes. The results are also to inform the pathways of impact and obtain greater insight into the patterns and correlates of child development in Indonesia. In order to understand the pathways not just at a household, but also a community level, the evaluation makes use of six questionnaires covering the state of children’s development and the context in which children develop. At baseline, there were six separate questionnaires: a child questionnaire that included child tasks, caregiver questionnaire, household head questionnaire, village midwife questionnaire, posyandu (integrated child health services clinic) questionnaire and a village head questionnaire. The midwife and posyandu were included for the baseline data collection as these are publicly provided health services targeted at young children and they are often the most common form of ECED services in a village. For the midline data collection, the midwife and posyandu questionnaires were replaced with a dusun level questionnaire related to project services (in treatment areas), and a village level questionnaire related to ECED services (in treatment and control areas). The midwife and posyandu questionnaires were dropped mainly for cost reasons. While the information from these service providers was valuable, the research team found it more beneficial to gather data on project implementation and more comprehensive information on ECED service availability and provision, and it was not feasible to have four village level questionnaires in the midline data collection.

#### Instrument development and piloting

Access to services, service utilization, asset inventory, dwelling attributes and consumption history by the household were collected using a series of questions that were previously constructed for the Indonesian Family Life Survey (IFLS), a renowned panel study focusing on demographic, poverty and health measures. The child and caregiver questionnaires include several internationally-recognized standard scale instruments and anthropometric measures. We also collected information about the child’s development and health history from the parent/caregiver. None of the standard tools used to measure childhood development had been applied in Indonesia before. As such, the instruments had to be translated and adapted to the Indonesian culture and circumstances, and the study design meant that some of the standard measures of child development needed to be amended.

#### Early Development Instrument (EDI)

The Early Development Instrument (EDI) is generally completed for each child in the kindergarten classroom by the child’s teacher. In Indonesia, very few children attend school at the age of 4 years, particularly in poor regions, and thus the first variation was that the EDI was to be completed by an interviewer with the caregiver of the child. The full EDI includes a core checklist of 104 items, combining five major areas of child development and 16 sub-domains. However, the EDI also comes in a short form with 48 items. Due to the number and length of instruments being used in the impact evaluation and concern for respondent burden, it was decided to use the short form of the EDI from inception. It is acknowledged that the author of the EDI does not recommend the use of the short form EDI prior to analyses of all the items by piloting the full EDI in a country first [34].

#### Strengths and Difficulties Questionnaire (SDQ)

The Strengths and Difficulties Questionnaire (SDQ) [35], which is also an informant-based assessment of a child, is also used internationally. The SDQ had already been translated to Indonesian by Wiguna and Hestyanti [36]; however, no validation data for the translated version is published.

#### Dimensional Change Card Sort (DCCS)

As a test of executive cognitive functioning, we included the Dimensional Change Card Sort (DCCS) task [37,38]. This task involves a child sorting cards with pictures that vary by shape and color into two groups according to one dimension (by shape), and then switching to sorting them by the other dimension (by color). Traditionally the colors are red and blue and the shapes represent a rabbit and a boat. For the purposes of the ECED impact evaluation we changed the shapes to a motorbike and a cat as these are more familiar to children in Indonesia.

#### Child tasks

Various tasks similar to those used in the Ages and Stages Questionnaire (ASQ) [39] and an instrument used in the Philippines for another World Bank project [31] were also included in the ECED impact evaluation. These included fine motor skills tasks (threading string through a bead), gross motor skills (sitting through to walking backwards, hopping on one foot and throwing a ball overhead), cognitive assessment (by drawing a circle, a person and a house), and expressive and receptive language (by identifying pictures and parts of their body).

#### Content validity and piloting of the instruments/questionnaires

Content validity was established over a period of 4 years from 2006 to 2009 involving meetings, focus groups and various pilots in the field. Initial meetings were held with staff from the Government of Indonesia who had expertise in early child development. Translators were present at these initial meetings. No modifications to the instruments were made at this stage other than the inclusion of a set of questions regarding the child’s knowledge of Muslim prayers and religious practices. This version of the instrument was piloted in a mix of poor urban and rural villages near Majalengka (West Java) in March 2007, gaining a total of eight completed questionnaires.

The pilot revealed difficulties in the translation of response ranges (that is, ‘sometimes agree’ through to ‘sometimes disagree’) with a distinct preference for simple yes or no answers. The interviewers were government staff from the Department of Early Childhood (PAUD) within the MoNE and one experienced fieldworker who had worked on the IFLS and other significant surveys in Indonesia. There was much debate among the interviewers regarding the five ordered response categories and if it was possible to generate a range from the respondents or not, with the expected response in the Indonesian language being ‘can’ (bisa) or ‘cannot yet’ (tidak bisa). In addition, the interviewers were concerned about a strong social desirability response bias displayed by the child’s caregivers. After this pilot testing, the study team added a series of child tasks, fieldworker observations and repeat questions within the instrument suite to try to counter the social response bias and increase our ability to assess reliability. Further translations and back-translations were undertaken to improve the understanding/interpretability of the ranged response.

In September 2007, this amended full suite of instrumentation was piloted in Subang (Central Java) with 26 households. Similarly to the previous pilot, there was still difficulty with the ranged response categories. In addition, questions that required some degree of subjectivity in the answer seemed to be very difficult for the respondents (for example, ‘How would you rate your child’s overall social/emotional development?’). Such questions required greater guidelines and training for the interviewers.

#### Baseline pilot study

After another series of modifications, the instruments were again back-translated to English for checking before a final and comprehensive pilot. This baseline pilot was conducted in January 2009 in Yogyakarta and Kebumen in Central Java by the survey firm, Bina Karya, which was hired by the MoNE to carry out the baseline survey. This pilot surveyed 200 households (children, caregivers and household heads), 20 midwives or posyandu (integrated child health services clinic) heads and ten village heads. The purpose of this pilot was primarily to validate the instruments, especially for the children, to ensure that the instruments captured a range of child capabilities and did not pose ceiling or floor effects. Secondly, the pilot was to test the comprehension of survey questions by respondents and the overall flow of the questionnaire, and serve as an opportunity for Bina Karya to practice the logistics of implementing the survey.

### Quality control

#### Project quality control

Frequent project coordination meetings with government were and still are being conducted by the World Bank as standard practice with any loan to a country. In addition, the World Bank’s project management team based in the Indonesian office generally organizes two annual formal review sessions, each producing an *aide*-*mémoire* which documents the project’s accomplishments, challenges and proposed remedies. The project management team is in daily contact with the MoNE and makes frequent visits to oversee project implementation.

#### Baseline survey quality control

Fieldwork training took place in March 2009 over 5 days in three locations throughout the country: Yogakarta (Central Java), Lampung (Sumatra) and Mataram (Lombok). Training covered the details of the fieldwork manual including protocols for obtaining consent, ensuring confidentiality, how to probe effectively and questionnaire content. The training also included a module for data entry staff to become familiar with the data entry program (the manual and program was developed by World Bank staff), and a field practice with live respondents.

Field teams used paper questionnaires but data were entered shortly after enumeration as data editors were co-located with enumerators during fieldwork. Baseline fieldwork took place from March to June 2009. Each field team consisted of five to six fieldworkers and a team supervisor. It was the supervisor’s responsibility to: gain support/permit from the village head, conduct the village level instruments (village head, midwife and posyandu), check all surveys for completeness and quality before leaving the village, and keep track of participation.

Baseline survey quality control was carried out by a combination of staff from the survey firm’s leadership team, the MoNE and the World Bank. Quality control during survey implementation included attending the pilot and training, frequent visits to the field to accompany supervisors and enumerators during questionnaire administration, and observing the process of data editing and entry. Survey firm supervisors and several MoNE and World Bank staff also revisited households to verify data collected by enumerators. Subsequent to fieldwork, MoNE and World Bank staff were involved in observing the second data entry and cleaning process which took place at Bina Karya offices in Jakarta. Throughout the entire survey effort, MoNE and World Bank staff provided feedback and recommendations to Bina Karya staff regarding measures to improve fieldwork management, interviewing quality, data entry processes, and clarifying instrument content.

Despite these quality checks and processes there were still problems with data quality. As an example, it was found that one fieldwork team entirely made up their data. Once this was discovered a new fieldwork team went back out to those villages and the data were collected properly.

#### Midline survey quality control

Many of the same procedures and protocols for the baseline were repeated for the midline data collection, with some exceptions. First, some questionnaire content was altered to reflect the baseline experience, for example, questions that were found to be ineffective, showing little variation or proving not to be understood by respondents were revised or dropped. The team also added a different suite of questionnaires at the village and dusun levels related to project implementation and overall ECED service provision and availability. Second, the MoNE implemented the survey differently. The MoNE recruited a team of five highly experienced survey consultants to lead the survey, along with an event organizing firm, which handled all administration and logistics of the survey, but not survey content or quality. The lead consultants were responsible for recruiting all fieldworkers and delivering high quality data.

Because of the changes to questionnaire content and implementing structure, two pilots were conducted prior to midline survey. The first pilot was conducted in Lombok in April and May 2010, and the second in Subang (West Java) in June 2010. The purpose of the pilot was similar to that of the baseline pilot, with a focus on the new questionnaires about ECED service provision and project implementation. The team conducted two pilots because the objective of the first was to test questionnaire content and served to refine the questionnaires, whereas the second pilot was to practice logistics of survey administration and get final estimates on survey administration time.

Midline survey training began with a 2-day workshop for district coordinators in Jakarta, followed by 7 days of fieldworker training in Bandung in late June/early July (thus all fieldworkers were trained together). Midline data collection took place in July and August 2010. Midline data collection quality control was largely similar to the baseline with survey team leadership, MoNE and World Bank staff making frequent field visits throughout the duration of the fieldwork to verify data collected and entered, accompany fieldworkers during interviews, and offer coaching and feedback regarding questionnaire administration and content. As with the baseline, a first round of data entry took place in the field with the second round of data entry in Jakarta.

### Planned analyses

As noted above, there are several hypotheses under the trial. One of the risks when collecting a large set of outcome variables is to highlight false positives in the final analysis, that is, significant coefficients are identified in the final analysis when, in fact, these could be due to natural randomness. To reduce this risk, for each hypothesis below we list (in bullets) which outcome variables we will use to test our research hypotheses. We believe that by limiting the analyses to a parsimonious set of variables defined *ex ante*, the risk of false positives is greatly reduced. The hypotheses under the trial are whether the ECED program has led to:

1) Greater access to ECED services

• The number of children aged 0 to 6 years in the village/ECED centers in the village

2) Higher participation rate in ECED services

• The fraction of children that attend an ECED service at least once a week by age five years

3) Higher enrolment rate in school at earlier ages

• The fraction of children aged 6 to 8 years that enrolled in primary school at the age of 6 years. (For children aged 7 and 8 years, their age of enrolment will be inferred by the grade they are in at the time of the survey)

4) More ‘school ready’ children

• The summary values of the EDI in the domains of physical health and well-being, social competence, emotional maturity, language and cognitive skills, communication skills, and general knowledge

• Mean score on the SDQ in the areas of emotional symptoms, conduct problems, hyperactivity/inattention, peer relationship problems and pro-social behavior

• The average numbers of stages children pass on the DCCS task (there are three stages)

• The fraction of tasks children can perform in the areas of gross motor skills, fine motor skills, language skills, cognitive skills and socio-emotional skills

5) Higher community awareness about the importance of ECED

• Parental knowledge about the location of key ECED services in the village

6) Higher persistent breastfeeding rates, improved nutrition and improved early childhood stimulation.

• Average number of months of exclusive breastfeeding for children when they were below the age of 6 months

• Height-for-age z-score, weight-for-height z-score

• Number of activities children engaged in last week (out of book reading, storytelling, drawing, music, playing with toys and helping in household chores) and fraction of children that play outside

The first steps in the analysis will be to further validate the instruments used. This will be undertaken by assessing floor and ceiling effects, correlates between measurements, and investigating gradients with socio-economic indicators as well as with age and gender. These analyses will also provide a unique snapshot of the early development of a large cohort of Indonesian children [40].

Having two control groups, an experimental and a matched control, is an unusual feature of this evaluation. Usually an experimental design would be the first choice, and the entire sample would be allocated to this. This rule of thumb was not followed for this evaluation because the effect of the intervention is expected to depend on the duration of exposure to the project. The largest measurable impact is thus expected for children that live in Batch 1 villages at the end of the project. Measuring this impact is the main objective of the evaluation. The randomized control sample is of limited value in estimating this impact as it will have already received the intervention by the end of the project. The matched control sample, on the other hand, will never receive the intervention. Using the observations from the Batch 1 villages and matched control villages, the impact of the project can be estimated using:

(1) $$Y_{\mathrm{ijt}}=\alpha_{t}+X_{\mathrm{ij}0}\beta_{t}+\delta_{t}T_{j}+\epsilon_{\mathrm{ijt}}$$

Where *i* denotes the child, *j* the village, and *t* = 1,2 indicates the time of the baseline, midline and endline surveys. *X* is a vector of village and child baseline characteristics, and *T* indicates whether the village received the project (*T* = 1 for Batch 1 villages and *T* = 0 for matched control villages).

This estimate is a valid impact estimate under the assumption that the outcomes at endline, given baseline characteristics, would be equally distributed in the Batch 1 villages and matched control villages in the absence of the intervention. This assumption cannot be tested at baseline but can be tested at midline. At midline, the averages observed in the randomized control sample of Batch 3 villages are a valid estimate of the expected outcomes in Batch 1 villages in the absence of the intervention. Whether these estimates are equal to those of the matched control sample can be tested by estimating:

(2) $$Y_{\mathrm{ij}1}=\gamma +X_{\mathrm{ij}0}\theta +\mathrm{\pi M}C_{j}+\epsilon_{\mathrm{ij}}$$

An estimate of *π* that is not significantly different indicates that the matched control sample would have been a valid control group at midline when using the sample of matched control (*MC* = 1) and randomized control villages (*MC* = 0). If this is true, it also increases the ‘comfort factor’ of making this assumption at endline. If not, it provides an indication of the direction in which the estimates from (1) will be biased.

At midline, the experimental design makes it possible to estimate the average treatment effect of the first 18 months of intervention with minimal assumptions. Batch 3 villages will still be a valid control group. Using data from Batch 1 (*T* = 1) and Batch 3 (*T* = 0), the average treatment effect can be estimated by:

(3) $$Y_{\mathrm{ij}1}=\rho +X_{\mathrm{ij}0}\varphi +\vartheta T_{j}+\epsilon_{\mathrm{ij}}$$

Outcomes will be indicators of child development and utilization of early childhood development services. The latter is of interest as the projects aims to increase child development through improved access to early childhood development services. Considering both types of outcome variables provides an opportunity to evaluate the pathways of impact.

#### Differential treatment effects

We will report results for the following sub-groups: boys/girls, poor/rich (defined on the basis of an asset index with poor being defined as those who are in the bottom half) and enrolment in ECED services before commencement of the project. All sub-groups will be defined on the basis of information collected in the baseline data.

#### Deviations from planned design and consequences for analysis

As mentioned earlier, one deviation from the research design was that the randomized assignment to Batches 1 and 3 was not adhered to. This happened in our case for 20 out of 310 villages. Because this misallocation occurred in relatively few villages, these can be accommodated by using an instrumental variable (IV) estimator in (3). In this case, *T*_*j*_ in (3) is replaced with the actual batch, or the time period of the actual intervention at the time of the survey, and is instrumented with *T*_*j*_. A comparison between the non-instrumented estimate and the IV estimate is insightful in this case. To estimate the impact of the entire project at endline using (1), there is only the non-experimental estimator available. The deviation between the non-instrumented estimator and the IV estimate will give an indication of the effect of using a non-experimental estimator on the parameter estimate.

Another significant deviation from the research design was that the baseline data collection did not take place before the Batch 1 villages started implementing the project. The baseline data collection took place on average 6 months after Batch 1 villages started implementation. Further, the midline data collection took place an average of 9 months after Batch 3 villages started implementation. The delays were a result of the MoNE’s prolonged procurement process to engage the survey fieldwork company and a desire to dispense the funds to the communities. Given these timing issues, in order to assess the impact of the project we will construct measures of dosage, such as months the project was active in a given village.

The consequence of the late baseline survey is that the pre-intervention values cannot be observed from the baseline data for Batch 1 villages. For Batch 3 villages, the intervention had not occurred at the time of the baseline. This is particularly problematic for pre-intervention levels of outcome variables, which in equations (1), (2) and (3) would normally be included in *X*_*ij*0_. With a late baseline, these outcome variables could already have been influenced by the intervention, thus leading to correlation between *X*_*ij*0_ and *T*_*j*_, also in the experimental design.

Our approach to manage this issue is two-fold. For the utilization variables, which we believe responded quickly to the opening of early childhood development services, we intend to reconstruct a variable of utilization before the start of the project based on retrospective data collected in the baseline survey. For child development variables, we intend to use the baseline data to estimate the impact of the intervention after 6 months comparing Batch 1 to Batch 3 villages.

The consequence of the late midline survey is more limited. When conducting the midline analysis in (3), the comparison is no longer between 18 months of implementation and no project, but rather between 20 and 9 months of implementation. This is because project implementation was delayed in Batch 3. By the time the midline data were collected, Batch 3 villages had been implementing the intervention for only 9 months. Therefore, we intend to use the midline data to estimate the impact of the project after Batch 1 villages have received the project for 20 months and Batch 3 villages have received the project for 9 months, that is, the impact of 11 months of differential exposure. For outcome variables which respond quickly to the treatment, such as utilization, this is a problem as both Batch 1 and Batch 3 villages received treatment. Our strategy will be to focus on utilization variables which capture utilization over the entire evaluation period, not only at the time of the survey. For outcome variables which respond more slowly to the treatment, such as child development outcomes, we simply report the comparison as above, indicating that this is the impact of the 18-month difference in treatment duration.

## Consent

Consent was requested by the fieldwork supervisors from the village head prior to commencing fieldwork. Once approval was provided at the village level, enumerators went through extensive confidentiality briefings with potential respondents and obtained verbal consent prior to commencing interviews.

### Participant incentives

#### Cluster level (village)

No incentives were provided to the village for participating, but villages were strongly encouraged by the MoNE, in the form of a letter that the fieldworkers carried with them, to participate in the study.

#### Individual level (household)

For both rounds of data collection, respondents were only offered a ‘gift’ after the interview was complete. This was done in an attempt to minimize the response bias that might have occurred had gifts been offered upon commencing the interview. For the baseline data collection, respondents were given small towels or pencils for participation. For the midline, respondents received 30,000 Indonesian rupiah (approximately US$3).

### Ethics approval

Survey studies conducted in Indonesia do not require ethics approval. As such, neither the MoNE nor the World Bank required ethics approval for this study, but the MoNE and the survey firm undertook several measures to ensure that districts, villages, dusuns and households were aware of the legitimacy of the study, and that the survey work did not interfere with local governance or community activities. With a letter of approval from the MoNE (Director General of Non-Formal and Informal Education, Direktorat Jenderal Pendidikan Non Formal dan Informal, Kementrian Pendidikan Nasional), the survey firm applied for a national permit from the Director General of National Unity and Politics, Ministry of Internal Affairs (Direktorat Jenderal Kesatuan Bangsadan Politik, Kementrian Dalam Negeri). The MoNE then issued a letter to each survey district education office requesting the survey firm’s permission to conduct the survey over a specified time period. The survey firm would show this letter from the MoNE to the district education office, which would issue a permit letter to the survey team, which could be presented to the village and dusun offices to gain permission to survey in these areas.

This process was repeated for the midline data collection and will need to be repeated prior to the endline data collection.

### Study governance

The study was originally lead and designed by MP and then taken over by AB (June 2008 to November 2010). From November 2010 to January 2012 the study was coordinated by JP and since that time has been coordinated by AH. During this time MP, AB, JP and AH were employed as full-time staff or consultants at the Indonesian office of the World Bank. Although strongly supported by the World Bank, the responsibility for the impact evaluation lies with the MoNE as committed in the loan agreement between the World Bank and the Republic of Indonesia. This means that the MoNE was responsible for procuring and managing the baseline and midline survey firms. World Bank staff mainly provided technical assistance in design and implementation, and quality control for the data collection, and have been involved in data analysis with the MoNE team. SB was originally employed as a consultant to the World Bank to help support the study design and to compile and pilot the instrumentation. SB, MP and AB were successful in an Australian Agency for International Development (AusAID) research grant to help facilitate the coordination of the study group, and to employ local early to mid-career academics to build capacity in evaluation and early child development in Indonesia. This grant employs both AM and ES, both based at the University of Gadjah Mada in Yogyakarta.

### Project timetable

The timeline of impact evaluation milestones are presented in Table 1.

**Table 1:** Timeline of impact evaluation milestones

**Impact evaluation milestones**	**Anticipated**	**Actual**
Sample size calculations	December 2006	December 2006
Randomization of 20 villages/districts to T and C status	March to July 2007	March to July 2007
Selection of matched controls	March to July 2007	March to July 2007
Discovery of randomization compliance issues	N/A	December 2008
Batch 1/treatment receives block grants	February 2008	November to December 2008
Batch 1/treatment opens services	December 2008	March 2008 to December 2009
Baseline survey	January 2008	March to June 2009^a^
Batch 3/randomized control receives block grants	May 2009	November 2009 to June 2010
Batch 3/control opens services	July to August 2010	January 2010 to present
Midline survey	March 2010	July to August 2010
Endline survey	Planned for early 2013	February to July 2013

^a^Fieldwork in most districts took place in March and April 2009, with some exceptions. Data collection in Lombok went into June due to some delays in paying enumerators and because the sample was over double that of the other districts. Because it was discovered that data were falsified in Sarolangun and Bengkulu, the World Bank hired a team to re-administer surveys in Bengkulu in June 2009 and in Sarolangun from October to December 2009. *C* control, *T* treatment.

## Discussion

Given the voluntary, non-random district selection in participating in the evaluation, this study cannot be considered representative of the MoNE’s ECED project or Indonesia as a whole. Although the sample covers five islands and nine districts and provinces, the sample is not representative of Indonesia as a whole, since district selection was not carried out proportional to the population and beneficiary districts volunteered for the project. Thus, when considering scaling up this project across Indonesia, we need to be cautious when advising policymakers due to the limited representativeness of the project; and to be cautious about saying how Indonesian children perform, since this sample does not reflect Indonesian children as a whole.

However, although this evaluation is compromised on external validity, its strengths lie in its internal validity and its utilization of internationally comparable instruments. This impact evaluation employs a design that is internally valid, evidenced by the random assignment to treatment and control status. This means that by the midline, characteristics that differ between treatment and (randomized) control villages can only be attributed to the project and not any characteristics that differ by respondents.

In addition to external validity, the other caution we have relates to the length of time between when the treatment and randomized control villages received the project. Most research literature to support the benefits of early intervention and child development are long-term studies that in many cases track into adulthood [2,41,42]. The current planned follow-up time for this study is more limited, and showing an impact might be difficult. Initially, the design called for 18 months between baseline and midline as well as 18 months difference in exposure to the project. Due to a combination of lack of adherence to the original study design and planned schedule, the time difference between baseline and midline data collection ended up being around 14 months, while the difference in exposure to the project between Batch 1 and Batch 3 was around 11 months by the time of the midline data collection. This short time span is precisely why the evaluation employed an additional matched control group. The advantage offered by the matched control group is that it allows us to track children who were not exposed to the project for the full 3 years. However, the primary disadvantage with the matched control group is the threat to internal validity.

While the project allowed for the provision of both center-based and outreach services for children, in practice most service provision has been center-based (based on World Bank operational data indicating that only 38% of the centers have organized outreach activities). The block grant was able to be used to equip the centers with toys, learning materials, food supplements and play equipment. The funding was also to be used to hire two local community members associated with each dusun, where one was to be trained as an ECED teacher and the other a community development worker to conduct outreach activities. The community was expected to provide infrastructure for the ECED center. The project funding to communities allows for flexibility in mechanisms of service provision, but stipulates that the services be targeted to children aged 0 through to 6 years with a specific focus on children aged 2 to 4 years, and poor families. This degree of flexibility at a community level means that there may be a large degree of variation across villages, also making it difficult to calculate dose and thus impact.

In terms of the dose of ECED services, children in the study sample are likely to have differentiated exposure to the treatment. Our sampling strategy involved random recruitment of children from within two dusuns before it was known in which dusuns the new ECED services would be located. Children who reside in the two dusuns where the ECED project ended up being implemented are potentially more likely to benefit as they live closer to the ECED services. Additionally, children enrolled in the ECED service may also benefit more than a child living in the same dusun but not attending the ECED service. The distribution of benefits will depend on both the decisions of the household and the management of the ECED center. A household will decide to what extent to participate in outreach activities and to enlist their children in center-based activities. The management of the ECED center will decide on the mix of center-based and outreach activities, and if there is excess demand, selection of children into activities conducted at the ECED center (although in practice we have not observed selectivity even in the face of excess demand). The study questionnaires have been developed to collect as much information as possible to determine both attendance and frequency of attendance, but also reasons for participating (or not) in ECED services.

In conclusion this study has and will present some significant quality, analytical and methodological challenges. Many of these challenges are being addressed at present as the study team are designing the endline survey to be conducted in early 2013. Suggested strategies to help enhance our ability to draw conclusions from the study include increasing the duration of follow-up and collecting data for all children in the study villages through the schools rather than just following the sample cohort. Such strategies will be dependent on funding and support from the MoNE and the World Bank for ongoing commitment to the study.

## Trial status

Ongoing.

## Abbreviations

ASQ: Ages and stages questionnaire; AusAID: Australian agency for international development; CDW: Community development worker; CONSORT: Consolidated standards of reporting trials; DCCS: Dimensional change card sort; ECDU: Early childhood development unit; ECED: Early childhood education and development; EDI: Early development instrument; ICC: Intracluster correlation coefficient; IFLS: Indonesian family life survey; IV: Instrumental variable; MoNE: Ministry of national education; PAUD: Department of early childhood; RCT: Randomized controlled trial; SDQ: Strengths and difficulties questionnaire.

## Competing interests

The authors declare that they have no competing interests.

## Authors’ contributions

MP and SB designed the original study. MP undertook the sample calculations. SB and MP designed the original questionnaire suite, with SB primarily responsible for the child development measures. AB and SB tested and revised the full suite of questionnaires for both baseline and midline. AB supported the MoNE in coordinating and managing both baseline and midline data collections, and cleaning and preliminary analysis, with support from MP, SB, AM and ES. JD oversaw the continuation of the project, and now AH has succeeded JD as the current study coordinator and will be managing the endline data collection. All authors contributed to the writing of this paper.
